# Survival by Treatment Recommendation and Receipt Among Older Patients With Early-Stage Cervical Cancer

**DOI:** 10.1001/jamanetworkopen.2025.32206

**Published:** 2025-09-16

**Authors:** Ryan Suk, Yueh-Yun Lin, Sarah Dilley, Rasheeta Chandler, Ran Xiao, Hui Shao, Jessica Wells

**Affiliations:** 1 Nell Hodgson Woodruff School of Nursing, Emory University, Atlanta, Georgia; 2 Department of Health Policy and Management, Rollins School of Public Health, Emory University, Atlanta, Georgia; 3 Winship Cancer Institute of Emory University, Atlanta, Georgia; 4 Center for Health Data Science & Analytics, Houston Methodist Academic Institute, Houston, Texas; 5 Division of Gynecologic Oncology, Department of Gynecology and Obstetrics, School of Medicine, Emory University, Atlanta, Georgia; 6 Hubert Department of Global Health, Rollins School of Public Health, Emory University, Atlanta, Georgia; 7 Department of Family and Preventive Medicine, School of Medicine, Emory University, Atlanta, Georgia

## Abstract

**Importance:**

Cervical cancer remains a substantial public health concern among older women, particularly those who are beyond the age of routine screening. Understanding the survival benefits of treating early-stage cervical cancer in this population is essential for optimizing care and improving outcomes.

**Objective:**

To analyze the survival outcomes based on the receipt of treatment and treatment recommendations among patients with early-stage cervical cancer aged 65 years and older.

**Design, Setting, and Participants:**

This retrospective cohort study of patients aged 65 years and older with a first diagnosis of localized, microscopically confirmed invasive cervical cancer used data from 17 Surveillance, Epidemiology, and End Results (SEER) cancer registries in the US from 2000 to 2020. Data were analyzed from May 2023 to January 2024.

**Exposure:**

Receipt of recommended treatment, specifically surgery and/or radiotherapy, as recorded in the SEER database. Treatment modalities were categorized based on recommendation status and actual receipt.

**Main Outcomes and Measures:**

Survival outcomes based on treatment recommendation and receipt status by treatment type expressed as (1) 5-year relative survival rates, (2) cervical cancer-specific mortality, estimated using competing risks models, and (3) adjusted hazard ratios (AHRs) calculated using Fine-Gray competing risk regression.

**Results:**

Among 2236 females included in the study, 66.3% (1482) were aged 65 to 74 years, 25.3% (565) were 75 to 84 years, and 8.4% (189) were aged 85 years or older. In the group aged 65 to 74 years, those who received surgery had significantly higher 5-year survival rates (91.2%, 95% CI, 88.4%-93.4%) compared with those not recommended for surgery (69.6%, 95% CI, 62.8%-75.4%) or those who were recommended but did not receive surgery (52.3%, 95% CI, 24.2%-74.3%). Similar patterns were observed in the group aged 75 to 84 years, in which receiving surgery was associated with higher survival rates (88.6%, 95% CI, 79.8%-93.7%). In multivariable analyses, receiving surgery (AHR, 0.28; 95% CI, 0.16-0.50) and radiotherapy (AHR, 0.48; 95% CI, 0.26-0.87) were significantly associated with lower cervical cancer–specific mortality compared with not receiving the recommended treatment.

**Conclusions and Relevance:**

In this cohort study, receipt of recommended treatment was associated with higher survival rates among older patients with early-stage cervical cancer. Addressing barriers to treatment adherence and improving early detection and preventive measures in this older population may enhance population health and reduce cervical cancer mortality in the aging population.

## Key Points

**Question:**

What are the survival differences by treatment recommendation and treatment receipt among patients with early-stage cervical cancer aged 65 years or older?

**Findings:**

In this cohort study of 2236 patients with early-stage cervical cancer aged 65 years and older, receiving recommended surgery or radiotherapy was significantly associated with a lower risk of cervical cancer–specific mortality compared with refusing or not receiving recommended treatment, after adjusting for other treatment factors and the number of cancer diagnoses.

**Meaning:**

In this study, older patients with localized cervical cancer who received recommended treatments experienced substantial survival benefits.

## Teaser

This cohort study assesses survival outcomes based on the receipt of treatment and treatment recommendations in patients aged 65 years or older with early-stage cervical cancer.

## Introduction

Cervical cancer remains a substantial public health concern despite advances in screening methods, the widespread availability of vaccination against human papillomavirus (HPV), and tools to treat precancerous lesions.^1^ Current US Preventive Services Task Force screening guidelines for women aged 21 to 65 years have been effective in reducing the incidence and mortality rates of cervical cancer among women of average risk.^2^ However, some have missed the opportunity to treat precancerous lesions before 66 years of age and continue to be at risk of developing invasive cancer after the screening eligible age.^3^ In fact, a second incidence peak for cervical cancer occurs between 60 and 70 years of age,^4^ with 20% of cervical cancers diagnosed after 65 years of age.^5^ The older population also represents 53% of cervical cancer deaths.^6^ From 2001 to 2017, the incidence rate by age group was higher in older women, with rates of 11.0 per 100 000 in those aged 65 to 74 years and 8.9 per 100 000 in those aged 75 years or older compared with 6.2 per 100 000 in women younger than 50 years.^7^ Additionally, the majority of diagnosed cervical cancer cases in the older age group are more likely to be detected at an advanced stage, complicating treatment and prognosis. This underscores a critical gap in cervical cancer early detection and prevention in this older age group.^8,9,10^

Treatment options for cervical cancer are determined by cancer location, type of cancer, and overall health. Localized treatment can include surgical procedures (cervical conization, simple hysterectomy, radical hysterectomy) or concomitant chemotherapy and radiation therapy. Treatment recommendations and practices for older patients with cervical cancer can differ from those for younger patients due to various factors, such as overall functional status, medical comorbidities, anatomical differences of postmenopausal women, and increased risk of potential complications from invasive treatments.^11,12^ Older patients with good overall health and longer life expectancy may benefit from more aggressive management, whereas those with substantial comorbidities or frailty might require a more conservative approach.

Although prior studies have documented survival differences based on treatment refusal in some types of cancers, these analyses typically include wide age ranges and mixed stages at diagnosis.^13,14,15,16,17,18^ Evidence remains limited for early-stage cervical cancer in older patients—a group for whom treatment can be curative but may not be offered or may be declined due to medical, personal, or social factors. Understanding the survival benefits of treating older patients with cervical cancer, particularly at an early stage, is therefore crucial. Evaluating how survival outcomes differ between those who received recommended treatments and those who did not can inform efforts to refine cervical cancer prevention and control strategies for this older age group. By focusing on early-stage cervical cancer, we aim to investigate how early detection and adequate treatment contribute to improved life expectancy among older patients. Therefore, we analyzed survival outcomes based on the receipt of treatment and treatment recommendations in patients aged 65 years or older with early-stage cervical cancer, a population that has largely aged out of screening eligibility but remains vulnerable to disease progression and treatment disparities.

## Methods

### Data Source and Population

We analyzed patients with localized cervical cancer diagnosed between 2000 and 2020 from 17 Surveillance Epidemiology and End Results (SEER) cancer registries (including Alaska Native Tumor Registry; Atlanta [Metropolitan]; California excluding San Francisco, San Jose-Monterey, and Los Angeles; Connecticut; greater and rural Georgia; Hawaii; Iowa; Kentucky; Los Angeles; Louisiana; New Jersey; New Mexico; San Francisco-Oakland; San Jose-Monterey, Seattle-Puget Sound; and Utah).^19,20^ The SEER 17 dataset consisted of cancer statistics, including patient demographics, cancer characteristics, treatment information, and follow-up outcomes, submitted by cancer registries to the National Cancer Institute. We used the data submitted in November 2022, which comprised 26.5% of the US population based on the 2020 Census conducted by the US Census Bureau.^21^ Per Emory University policy, this study was exempt from institutional review board approval and informed consent requirements because deidentified data were used. The study follows the Strengthening the Reporting of Observational Studies in Epidemiology (STROBE) reporting guideline.^22^

We included patients categorized as female in the registry who were aged 65 years or older when diagnosed with a first invasive primary cervical cancer at the localized stage. The *International Classification of Diseases for Oncology*, *Third Edition* (ICD-O3), site codes C53.0 to C53.9, and histology codes 8010 to 8671 and 8940 to 8941 were used to define cervical cancer diagnosis. All cases of cervical cancer included in this study were malignant and microscopically confirmed. The localized stage was identified using the SEER Combined Summary Stage, which includes the International Federation of Gynecology and Obstetrics stages IA1, IA2, IB, and I (not further specified).^23^ To include a sufficient sample size, maintain consistent age intervals, and capture age-relative differences in cancer survival, age at diagnosis was grouped into 65 to 74 years, 75 to 84 years, and 85 years or older. Patients of unknown age, age at diagnosis older than 99 years, alive without survival time, and missing values in the survival table were excluded from the analysis (eFigure in Supplement 1).

In the SEER database, treatment modalities were coded based on their recommendation and actual receipt statuses. For surgical interventions, classifications included: surgery performed, surgery not recommended, surgery recommended but unknown if performed, surgery recommended but not performed, and unknown. Radiotherapy was categorized as performed, not performed or unknown, recommended but unknown if performed, and refused. The study did not include chemotherapy as a primary independent variable but only as a control in the multivariable regression models due to the database providing only procedural status (yes vs no or unknown) without recommendation specifics.

### Statistical Analysis

We first estimated the 5-year relative survival rates by treatment recommendations and receipt of procedure using the 60 monthly intervals precalculated from the complete date of diagnosis and the date of death or last follow-up.^24^ To estimate the relative survival, observed survival was compared with expected survival in the general US population, assuming independent competing death causes using the actuarial method and Ederer II method for expected survival adjusted for patient demographics.^25,26^ Calculations accounted for patients at risk only until death or follow-up loss, censoring data at 99 years or December 31, 2020, for those alive, lost to follow-up, or dead from other causes.

To further assess the association of cervical cancer mortality risk with treatment recommendation and treatment receipt status, we used an analytical approach that accounts for competing risks from other causes of mortality. We first estimated cumulative risk rates by treatment status using the competing risk model and the Gray test in the single-factor analysis to identify initial differences in mortality risk associated with treatment status while considering the presence of competing risk.^27^ Additionally, we used a multivariable analysis with the Fine-Gray competing risk regression model to assess the cervical cancer mortality risk and associated factors. The results are expressed as adjusted hazard ratios (AHR), which quantify the association of each variable with the subdistribution hazard of cervical cancer mortality risk adjusted for covariables. The covariables used in the multivariable model included surgery status, radiation status, chemotherapy status, and the number of primary cancer diagnoses.

Data were analyzed from May 2023 to January 2024. All tests were 2-sided, and *P* < .05 was considered statistically significant. We used SEER*Stat, version 8.4.2 (National Cancer Institute), SAS, version 9.4 (SAS Institute), and R, version 4.4.1 (R Core Team) for all analyses.

## Results

Among 2236 females included in the study, 66.3% (1482) were aged 65 to 74 years, 25.3% (565) were 75 to 84 years, and 8.4% (189) were 85 years or older. Among the group aged 65 to 74 years, 76.4% (1132) received surgery, 1.3% (19) were recommended but did not receive surgery, and 21.5% (318) were not recommended to receive surgery. For radiotherapy, 41.4% (613) received radiotherapy and 56.0% (830) did not receive radiotherapy, and 1.4% (21) refused the recommended radiotherapy. In this age group, 27.1% (401) received chemotherapy. In contrast, among the 189 patients aged 85 years or older, a lower percentage of patients were recommended or received surgery; 34.4% (65) received surgery, and 4.8% (9) were recommended but did not receive surgery. Surgery was not recommended in 56.6% (107) of individuals in this age group. In this group, 52.9% (100) received radiotherapy, 41.8% (79) did not receive radiotherapy, and 4.8% (9) refused to receive the therapy. Only 12.7% (24) of individuals in this age group received chemotherapy (Table 1).

**Table 1.** Baseline Treatment Status by Age Group

Treatment status	Patients, No. (%)			
	Overall (N = 2236)	Aged 65-74 y (n = 1482)	Aged 75-84 y (n = 565)	Aged ≥85 y (n = 189)
Surgery status				
Recommended, not performed	47 (2.1)	19 (1.3)	18 (3.2)	9 (4.8)
Not recommended	635 (28.4)	318 (21.5)	210 (37.2)	107 (56.6)
Performed	1528 (68.3)	1132 (76.4)	331 (58.6)	65 (34.4)
Recommended, status unknown	16 (0.7)	9 (0.6)	4 (0.7)	3 (1.6)
Unknown	10 (0.5)	4 (0.3)	2 (0.4)	5 (2.6)
Radiotherapy status				
Recommended, patient refused	39 (1.7)	21 (1.4)	9 (1.6)	9 (4.8)
Performed	1006 (45.0)	613 (41.4)	293 (51.9)	100 (52.9)
Not performed or unknown	1165 (52.1)	830 (56.0)	256 (45.3)	79 (41.8)
Recommended, status unknown	26 (1.2)	18 (1.2)	7 (1.2)	1 (0.5)
Chemotherapy				
Not performed or unknown	1668 (74.6)	1081 (72.9)	422 (74.7)	165 (87.3)
Performed	568 (25.4)	401 (27.1)	143 (25.3)	24 (12.7)

**Figure 1.**
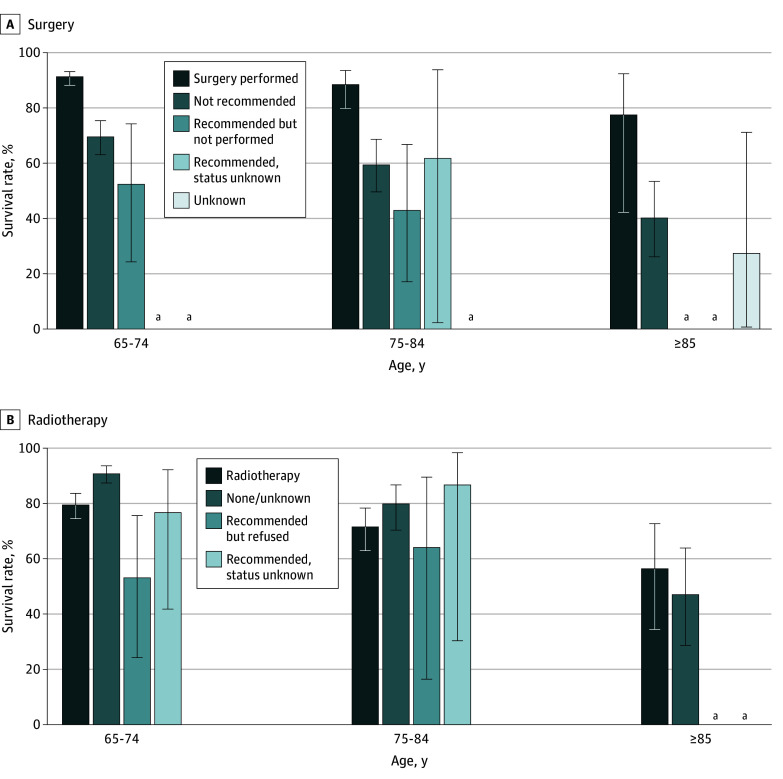
Five-Year Relative Survival Rates by Treatment Recommendation and Receipt Status ^a^The statistic could not be calculated.

Figure 1 and the eTable in Supplement 1 present the age group–specific 5-year relative survival rates by treatment status. For surgical treatments, patients who received surgery had a significantly higher survival rate (91.2%; 95% CI, 88.4%-93.4%) compared with those who were not recommended surgery (69.6% 95% CI, 62.8%-75.4%) or those who were recommended but did not receive surgery (52.3%; 95% CI, 24.2%-74.3%) within the group aged 65 to 74 years. Receiving the recommended surgery also resulted in a significantly higher survival rate (88.6%; 95% CI, 79.8%-93.7%) than not receiving recommended surgery (42.7%; 95% CI, 16.7%-66.8%) among those aged 75 to 84 years, while no statistically significant difference was seen among those aged 85 years or older. Compared with patients who received radiotherapy (79.7%; 95% CI, 75.1%-83.6%) or those who refused the recommended radiotherapy (53.2%; 95% CI, 24.0%-75.7%), patients who did not receive radiotherapy (or receipt was unknown) had a significantly better survival rate (91.0%; 95% CI, 87.6%-93.5%) in the group aged 65 to 74 years. For the other age groups, the observed differences were not statistically significant.

**Figure 2.**
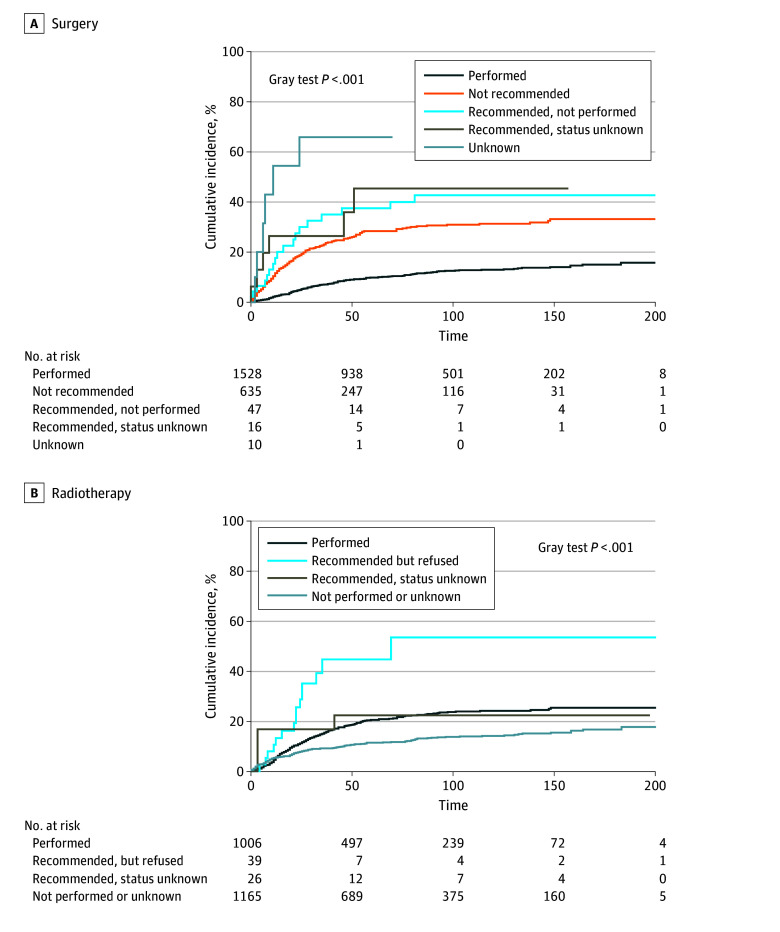
Cumulative Incidence of Cancer-Specific Death by Treatment Recommendation and Receipt Status

We also visualized the cumulative cervical cancer–specific mortality using a competing risks model (Figure 2). The Gray test, which was *P* < .001 for comparisons by both surgery and radiotherapy status, indicated statistically significant differences in cumulative incidence functions between the different strata.

In the multivariable Fine-Gray competing risk model (Table 2), receipt of surgery was associated with a significantly decreased risk of cervical cancer mortality (AHR, 0.28; 95% CI, 0.16-0.50) compared with not undergoing recommended surgery. When stratified by age groups, this association continued to be significant for the groups aged 65 to 74 years (AHR, 0.37; 95% CI, 0.16-0.86) and 75 to 84 years (AHR, 0.20; 95% CI, 0.09-0.44). The receipt of radiation therapy (AHR, 0.48; 95% CI, 0.26-0.87) and not receiving radiation therapy (AHR, 0.42; 95% CI, 0.23-0.75) were both associated with a significantly decreased risk of cervical cancer mortality compared with refusal of the recommended therapy. In the group aged 65 to 74 years, those who underwent radiotherapy (AHR, 0.41; 95% CI, 0.17-0.99) and those who did not (AHR, 0.24; 95% CI, 0.10-0.58) had a significantly lower rate of cervical cancer mortality than those who declined the recommended therapy, maintaining a consistent trend. For all age groups, there was no association with the receipt of chemotherapy or the total number of primary cancer diagnoses.

**Table 2.** Mortality Associated With Treatment Status and Stratified by Age Group in a Multivariable Fine-Gray Competing Risk Model

Treatment status	Mortality risk by group, AHR (95% CI)			
	Overall	Age 65-74 y	Age 75-84 y	Age ≥85 y
Surgery status				
Recommended, not performed	1 [Reference]	1 [Reference]	1 [Reference]	1 [Reference]
Not recommended	0.81 (0.46-1.43)	0.83 (0.35-1.96)	0.59 (0.28-1.27)	1.91 (0.53-6.88)
Performed	0.28 (0.16-0.50)	0.37 (0.16-0.86)	0.20 (0.09-0.44)	1.20 (0.34-4.31)
Recommended, status unknown	1.37 (0.49-3.85)	2.02 (0.43-9.44)	0.93 (0.18-4.76)	2.02 (0.16-26.01)
Unknown	2.64 (0.91-7.62)	4.30 (0.94-19.67)	7.41 (2.65-20.72)	1.41 (0.18-10.87)
Radiotherapy status				
Recommended, patient refused	1 [Reference]	1 [Reference]	1 [Reference]	1 [Reference]
Performed	0.48 (0.26-0.87)	0.41 (0.17-0.99)	0.52 (0.12-2.23)	0.55 (0.19-1.62)
Not performed or unknown	0.42 (0.23-0.75)	0.24 (0.10-0.58)	0.65 (0.15-2.79)	0.93 (0.34-2.54)
Recommended, status unknown	0.77 (0.26-2.33)	0.85 (0.22-3.33)	0.48 (0.04-6.34)	NA
Chemotherapy				
Not performed or unknown	1 [Reference]	1 [Reference]	1 [Reference]	1 [Reference]
Performed	0.84 (0.63-1.11)	0.88 (0.58-1.34)	1.01 (0.63-1.62)	0.78 (0.32-1.89)
No. of cancers	0.82 (0.64-1.05)	0.95 (0.71-1.28)	0.87 (0.56-1.37)	NA

Abbreviations: AHR, adjusted hazard ratio; NA, not applicable.

## Discussion

Our findings provide insights into the survival rates associated with the management of localized cervical cancer in the older population. Notably, the highest 5-year survival rates were evident among those who were recommended treatment procedures, such as surgery or radiotherapy, and who subsequently underwent the procedure. However, a noteworthy difference was observed in patients who, despite receiving a treatment recommendation, did not undergo the procedure, with their survival rates being significantly lower. This observed discrepancy of older patients deemed suitable for surgery or radiotherapy who did not proceed with the recommended treatment raises vital considerations. Such a decision might arise from a myriad of factors, including possible shortcomings within the health care system, socioeconomic challenges, or personal choices rooted in cultural or individual values.^28,29,30,31^ It is critical to understand these underlying reasons to ensure every patient has equitable access to and receives appropriate care, especially when life-extending treatments are available.

Although prior studies in breast, lung, melanoma, and other cancers have documented similar worse outcomes among patients who refused recommended treatments, they typically spanned broad age ranges and mixed cancer stages.^13,14,15,16,17,18^ Studies also show that older patients are more likely to decline or not receive recommended treatments, with treatment refusal often associated with cancer stage and socioeconomic disadvantage.^13,15,16,17,18^ However, evidence specific to older patients and/or early-stage cancer is scarce. In this study, we focused specifically on patients aged 65 years or older with early-stage cancer—a group in which curative treatment is often possible but sometimes not offered or declined. We found that failure to receive recommended surgery or radiotherapy at this potentially curative stage was associated with significantly worse survival outcomes.

The group of patients not initially recommended for surgery exhibited survival rates that, although at higher point estimates, were not statistically significantly different from those who were recommended but did not undergo the procedure. This finding suggests that a variety of clinical factors may influence the initial treatment recommendations. Patients who were not recommended for surgery might have been diagnosed at an earlier stage or may have been considered unsuitable candidates for surgery due to comorbid conditions, frailty, or overall health status.^11,12^ These considerations highlight the complexity of clinical decision-making and the importance of individualized patient assessments in treatment planning. Moreover, the survival benefits observed with radiotherapy were evident among patients who were recommended and received the treatment compared with those who were recommended but refused to receive the treatment. In contrast, the high survival rates observed in those not recommended for radiotherapy suggest that their likely smaller, more operable tumors were adequately managed through surgery without requiring radiotherapy.^32,33^

These findings emphasize the need for more sophisticated decision aids for tailored treatment strategies that consider the unique needs and circumstances of older patients with cervical cancer.^34,35^ Moreover, potential clinician age bias may contribute to underrecommendation or less assertive recommendation of curative treatments in older patients with cancer,^36,37,38,39^ highlighting the need for more objective, structured geriatric assessment and decision aids. For clinicians, the challenge lies in balancing the potential benefits of aggressive treatments against the risks posed by comorbidities and overall patient frailty as well as patient preferences.^30^ Recognizing and addressing any age bias and barriers to receiving recommended care is crucial, as chronological age alone should not preclude older patients with cervical cancer from receiving appropriate, life-extending treatments.

Moreover, considering the substantial survival benefits associated with treating early-stage invasive cervical cancer, extending cancer screening efforts beyond the current age limits might be beneficial. The growing global population of older individuals presents new challenges and opportunities for health care systems in the era of longevity. As life expectancy increases, so does the likelihood of age-related health issues, including cancer. While cervical cancer incidence peaks at a younger age than other cancer types, many older individuals are still at risk of developing cervical cancer as they age,^5,6^ especially those with good overall health and longer life expectancy. In fact, the likelihood of developing invasive cervical cancer in individuals aged 65 to 84 years (1 in 564) is not markedly lower than in those aged 50 to 64 years (1 in 554) or younger than 49 years (1 in 337).^3^ Moreover, a substantial racial disparity has been reported; Black patients are more likely to be diagnosed after the age of 65 years, suggesting that current screening and prevention strategies may be insufficient in reaching this population.^40^ This highlights the potential need to explore personalized algorithms to optimize prevention and early detection strategies for this age group.

Future research should investigate the multifaceted factors contributing to treatment refusal and nonreceipt despite clinical recommendations as well as potential clinician bias in treatment decisions for older patients with cancer, even in early-stage cancer. These factors may include socioeconomic status, access to care, health system barriers, patient preferences, and broader social determinants of health. Additionally, efforts should focus on developing and effectively implementing tailored intervention strategies that address these diverse challenges to improve adherence to treatment protocols. Research should also address the potential benefits of extending screening strategies to older populations, aiming to prevent the progression of invasive cancer and improve overall survival rates. This is particularly significant, as cervical cancer screening rates are markedly declining in the US, including in those who never benefited from the HPV vaccine, with lack of knowledge being the most common reason for underscreening.^41^ Understanding such missed opportunities can guide future clinical practices to more effectively prevent cancer and improve health outcomes in the era of longevity.

### Limitations

This cohort study has several potential limitations that should be considered when interpreting the results. First, the data were derived from the SEER registry, which, while comprehensive, does not include detailed information on comorbidities or concurrent treatments. This lack of data on comorbid conditions limits our ability to fully understand the clinical reasons behind treatment decisions except for the recommendation status. Second, the registry does not capture the reasons behind treatment refusal or nonreceipt, which may include patient preferences, socioeconomic barriers, or health care system limitations. Third, the study does not account for quality-of-life measures or patient-reported outcomes, which capture nuanced patient perspectives. Also, with only 17 cancer registries included in this SEER dataset, the relatively small sample size of older patients with localized cervical cancer who refused treatment also limits the statistical power of the study, although the differences between those who received treatment were found to be significant. Fourth, for radiotherapy, the no or unknown category includes individuals who did not receive treatment as well as those with missing data on recommendation and receipt. While the unknown proportion is typically small, this coding does not fully distinguish between these statuses. Further studies incorporating comorbid conditions, reasons for recommended treatment nonreceipt, quality-of-life measures, and larger patient populations are needed to develop strategies to improve treatment adherence and outcomes, particularly in the early stages of cervical cancer when the benefits of treatment are most pronounced.

## Conclusions

Our cohort study highlights the significant survival benefits associated with the receipt of recommended treatments in patients aged 65 years or older with early-stage cervical cancer. These findings underscore the importance of early detection and the need for personalized treatment approaches that account for the health status, comorbidities, and individual preferences of older patients rather than chronological age. By addressing the barriers to treatment adherence and optimizing care strategies, we can improve the survival outcomes and overall management of cervical cancer in the aging population.

## Notes

Supplement 1. **eFigure.** Inclusion Flowchart **eTable.** Five-Year Relative Survival Rates by Treatment Recommendation and Receipt Status

Supplement 2.
Data Sharing Statement
