# Supplementary material for: Survival by Treatment Recommendation and Receipt Among Older Patients With Early-Stage Cervical Cancer
Source: JAMA Netw Open. 2025 Sep 16;8(9):e2532206. doi: 10.1001/jamanetworkopen.2025.32206 (PMC12441871; doi:10.1001/jamanetworkopen.2025.32206)
Supplement: Supplement 1. — eFigure. Inclusion Flowchart eTable. Five-Year Relative Survival Rates by Treatment Recommendation and Receipt Status [file jamanetwopen-e2532206-s001.pdf]

Supplemental Online Content

Suk R, Lin YY, Dilley S, Chandler R, Xiao R, Shao H, Wells J. Survival by treatment recommendation and receipt among older patients with early-stage cervical cancer. *JAMA Netw Open*. 2025;8(9): e2532206. doi: 10.1001/jamanetworkopen.2025.32206

eFigure. Inclusion Flowchart

eTable. Five-Year Relative Survival Rates by Treatment Recommendation and Receipt Status

This supplemental material has been provided by the authors to give readers additional information about their work.

© 2025 Suk R. et al. *JAMA Network Open*.

eFigure. Inclusion Flowchart

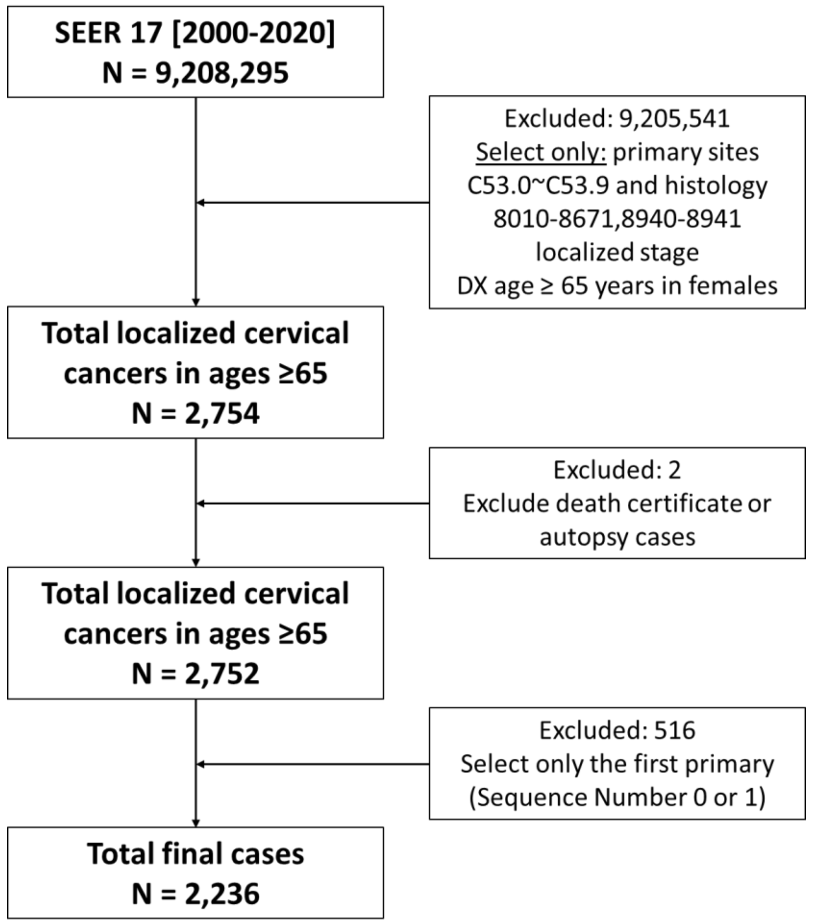

© 2025 Suk R. et al. *JAMA Network Open*.

eTable. 5-Year Relative Survival Rates by Treatment Recommendation and Receipt Status

	**65-74 years**	**75-84 years**	**≥85 years**
	Relative survival (95% CI)	Relative survival (95% CI)	Relative survival (95% CI)
**Surgery status**			
**Recommended, not performed**	52.30% (24.20-74.30)	42.70% (16.70-66.80)	0.00% (+)
**Not recommended**	69.60% (62.80-75.40)	59.60% (49.20-68.50)	39.80% (26.00-53.20)
**Performed**	91.20% (88.40-93.40)	88.60% (79.80-93.70)	77.60% (43.00-92.60)
**Recommended, status unknown**	+	61.60% (2.60-93.80)	0.00% (+)
**Unknown**	0.00% (+)	0.00% (+)	27.20% (0.70-71.20)
**Radiotherapy status**			
**Recommended, patient refused**	53.20% (24.00-75.70)	64.00% (16.50-89.50)	+
**Performed**	79.70% (75.10-83.60)	71.70% (63.20-78.50)	56.40% (35.60-72.70)
**Not performed/ unknown**	91.00% (87.60-93.50)	79.90% (70.40-86.70)	47.30% (28.60-63.90)
**Recommended, status unknown**	76.70% (42.20-92.20)	87.00% (29.50-98.40)	+

CI, Confidence interval; + The statistic could not be calculated.

© 2025 Suk R. et al. *JAMA Network Open*.
