# Supplementary material for: Survival by Treatment Recommendation and Receipt Among Older Patients With Early-Stage Cervical Cancer
Source: JAMA Netw Open. 2025 Sep 16;8(9):e2532206. doi: 10.1001/jamanetworkopen.2025.32206 (PMC12441871; doi:10.1001/jamanetworkopen.2025.32206)
Supplement: Supplement 2. — Data Sharing Statement [file jamanetwopen-e2532206-s002.pdf]

# Data Sharing Statement

Suk. Survival by Treatment Recommendation and Receipt Among Older Patients With Early-Stage Cervical Cancer. *JAMA Netw Open*. Published September 16, 2025. doi:10.1001/jamanetworkopen.2025.32206

**Data**
**Data available:** Yes
**Data types:** Other (please specify)
**Additional Information:** Data is publicly available through NCI SEER: https://seer.cancer.gov/seerstat/
**How to access data:** https://seer.cancer.gov/seerstat/
**When available:** With publication

**Supporting Documents**
**Document types:** None

**Additional Information**
**Who can access the data:** Data and data dictionary are all publicly available at: https://seer.cancer.gov/seerstat/
**Types of analyses:** For research purposes
**Mechanisms of data availability:** According to the NCI SEER data agreement.
